# mlCAF: Multi-Level Cross-Domain Semantic Context Fusioning for Behavior Identification

**DOI:** 10.3390/s17102433

**Published:** 2017-10-24

**Authors:** Muhammad Asif Razzaq, Claudia Villalonga, Sungyoung Lee, Usman Akhtar, Maqbool Ali, Eun-Soo Kim, Asad Masood Khattak, Hyonwoo Seung, Taeho Hur, Jaehun Bang, Dohyeong Kim, Wajahat Ali Khan

**Affiliations:** 1 Ubiquitous Computing Lab, Department of Computer Engineering, Kyung Hee University (Global Campus), Seocheon-dong, Giheung-gu, Yongin-si, Gyeonggi-do 446-701, Korea; asif.razzaq@oslab.khu.ac.kr (M.A.R.); sylee@oslab.khu.ac.kr (S.L.); usman@oslab.khu.ac.kr (U.A.); maqbool.ali@oslab.khu.ac.kr (M.A.); hth@oslab.khu.ac.kr (T.H.); jhb@oslab.khu.ac.kr (J.B.); dhkim@oslab.khu.ac.kr (D.K.); wajahat.alikhan@oslab.khu.ac.kr (W.A.K.); 2 School of Engineering and Technology, Universidad Internacional de La Rioja (UNIR), C/ Almansa 101, 28040 Madrid, Spain; claudia.villalonga@unir.net; 3 Department of Electronic Engineering, Kwangwoon University 20 Kwangwoon-ro, Nowon-gu, Seoul 01897, Korea; eskim@kw.ac.kr; 4 College of Technological Innovation, Zayed University, Abu Dhabi 144534, UAE; Asad.Khattak@zu.ac.ae; 5 Department of Computer Science, Seoul Women’s University, Seoul 01797, Korea; hwseung@swu.ac.kr

**Keywords:** context-awareness, ontologies, reasoning, fusioning, human behavior identification

## Abstract

The emerging research on automatic identification of user’s contexts from the cross-domain environment in ubiquitous and pervasive computing systems has proved to be successful. Monitoring the diversified user’s contexts and behaviors can help in controlling lifestyle associated to chronic diseases using context-aware applications. However, availability of cross-domain heterogeneous contexts provides a challenging opportunity for their fusion to obtain abstract information for further analysis. This work demonstrates extension of our previous work from a single domain (i.e., physical activity) to multiple domains (physical activity, nutrition and clinical) for context-awareness. We propose *multi-level Context-aware Framework* (mlCAF), which fuses the multi-level cross-domain contexts in order to arbitrate richer behavioral contexts. This work explicitly focuses on key challenges linked to multi-level context modeling, reasoning and fusioning based on the mlCAF open-source ontology. More specifically, it addresses the interpretation of contexts from three different domains, their fusioning conforming to richer contextual information. This paper contributes in terms of ontology evolution with additional domains, context definitions, rules and inclusion of semantic queries. For the framework evaluation, multi-level cross-domain contexts collected from 20 users were used to ascertain abstract contexts, which served as basis for behavior modeling and lifestyle identification. The experimental results indicate a context recognition average accuracy of around 92.65% for the collected cross-domain contexts.

## 1. Introduction

Context-awareness (CA) is considered to be an essential element in the ubiquitous and pervasive computing systems [1] and is widely recognized by the research community. It is treated as a key technology for situation awareness applications such as [2,3]. Dey et al. [4] defined the context as *“context is any information that can be used to characterize the situation of an entity”*. In the last two decades, significant work has been performed by developing CA prototypes, middleware, and applications [5]. CA provides a good approximation to the people for their interactions with the environment. State-of-the-art technologies play a critical role in capturing those interactions using several sensing modalities such as accelerometers or GPS sensors embedded in wearables and subsequently interpreted as *contexts*. However, it is challenging in context-aware infrastructure to exploit, integrate, and culture to enrich the fused information in the changing environment. It is pertinent to mention that most existing context-aware solutions cater contextual information in various discrete domains [6,7] and are unable to handle it cohesively. This discreteness provides enough opportunity to develop a context-based cross-domain fusion framework to model entities, dynamic relationships and realize richer human behavior information through fused outputs.

Context fusioning (CF) is considered as an integral part of any context-aware system [8]. For cohesive contextual information, the CF is deemed as a key approach to fuse various contexts from multiple domains [9,10] as well as to increase the understandability of a user’s context. CF helps to identify relevant actions, correlates their sequence in cross-domains [11]. It also handles the redundancy of contextual information for raising the context confidence [8]. Even though, the state-of-the-art CF approaches have gained much importance, however, they were unable to prove its deployment for real-world personalized user context in the health and wellness domain [8,12].

Keeping in mind the above-mentioned facts, our motivation was to design and develop an efficient framework to fuse the multi-levels cross-domain contexts for providing a richer contextual information. To achieve this goal, this study was undertaken with the following objectives: (1) extend the state-of-the-art context-awareness approaches from a single domain (i.e., physical activity) to cross-domains (i.e., physical activity, nutrition, and clinical) (see Section 3.2), (2) infer a more abstract representation of cross-domain contexts (see Section 5.1), (3) separate the ontology model (T-Box) and the application data (A-Box) (see Section 6.2), and (4) provide an effective contextual state of the users in a real-time manner to boost health and wellness services (see Section 6).

The key contributions of this research are: (1) enhanced physical activity domain ontology [6] with inclusion of nutrition (57 food items) and clinical (6 blood glucose and 5 blood pressure levels) domains; (2) functional framework for administering the *Web Ontology Language* (OWL) reasoning paradigm comprising of constraint-based rules (real-time) and *Semantic Query-Enhanced Web Rule Language* (SQWRL) based queries (offline) for recognizing contexts and behavioral patterns; (3) proof of concept for ontology modeling, framework implementation, and evaluation in health and wellness domains.

**Figure 1:**
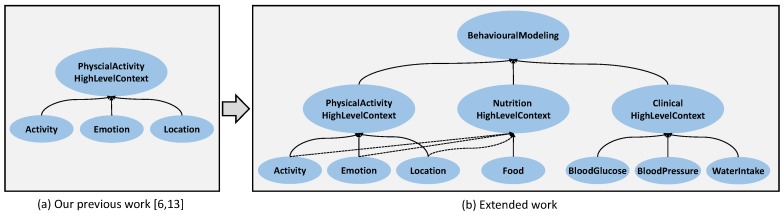
Extended Domains for Ontology evolution in mlCAF.

In this study, an open source efficient framework *multi-level Context-Awareness Framework* (mlCAF) is proposed, which fuses the multi-levels cross-domain contexts efficiently to provide a richer context-awareness information to its users. This study is the extension of our previous work [6,13], which is illustrated in Figure 1. Figure 1a indicates our previous work, which relies only on a single domain consisting of physical activities whereas, Figure 1b presents the extended version, which addresses challenges related to context-aware behavioral representation accurately for users by introducing cross-domain (i.e., physical activity, nutrition, and clinical) to support the health as well as the wellness domain.

The proposed framework under discussion is designed and developed for the Ubiquitous Computing Lab., Kyung Hee University, Yongin (http://uclab.khu.ac.kr/) project named *Mining Minds* (http://www.miningminds.re.kr/) (for details see Section 3), however, it can also be deployed with other platforms. The current version of this proposed framework is available for downloading from the GitHub open source platform [14].

For the realization of mlCAF, this study has considered diabetes scenario, as it has a strong relationship between physical activity, and dietary habits [15]. Usually, physical activities are recommended to people who suffer from diabetes as a part of a glycemic control and overall health improvement [15]. *Global Report on Diabetes* [16] by *World Health Organization* (WHO) states physical inactivity and unhealthy dietary practices are the key risk factors for this chronic disease. However, these key risk factors are modifiable through environmental, behavioral and lifestyle changes but they require raising people’s self-awareness.

The rest of the paper is organized as follows: Section 2 presents the related work. Section 3 introduces short descriptions of the *Mining Minds* platform, the evolved ontology illustrated with examples and the extended architecture. Section 4 provides insights into ontology-based context reasoning, and usage of semantic rules. Section 5 discussed mlCAF with example scenarios for *Vertical Fusioning* and *Horizontal Fusioning* techniques. Section 6 introduces the details of experimental results and evaluation along with behavioral rule definitions. Finally, main conclusions are drawn jointly with future steps in Section 7.

## 2. Related Work

Nowadays, the most dominant context information consumers are mobile applications, which engage different capabilities of sensors in the shape of context [11]. Sensor-equipped smart devices are widely used for providing health monitoring services [17]. These devices provide intelligence for recognizing daily life activities performed by users like standing, walking, driving etc. *Context-aware* (CA) systems from an *Internet of Things* (IoT) perspective were also surveyed [5] as for how CA can play its role in aggregating contextual information rather than processing whole sensory data. The work [18] introduced details of context-aware systems and their role in developing applications for end users. The recent advancements in IoT identify that context-awareness has become an inevitable part of IoT applications. All the information related to sensors not only, from a specific domain but also from heterogeneous domains, comes under the definition of context-awareness.

The heterogeneous domains handle dynamic contexts in a smart environment, to represent daily life activities correctly and assist people well-being with technologies [19]. The cross-domain context management can also play the vital role in knowledge integration from different sources in even recommender systems [20]. The initial efforts on cross-domain context management in *Feel@Home* environment [21] addressed context producer-consumer patterns for intra-domain and cross-domain in home/office environment by using indoor applications and executing mobile applications on mobile devices. However, this framework supported distributed administration and processing of contexts. The challenges linked to the cross-domain context management were also addressed by [22], in which authors identified context patterns in the multi-domain environment and proposed iCROSS context management infrastructure. Lin et al. [23] developed a unified approach for mutual tagging of heterogeneous domains constituted by the people, their associated events at certain locations by integrating based on feature similarity and cross-domain relations.

Context act as a key binding element for integrating information at different levels [9] in multi-domains. For instance, context information can be a valuable input taken as a parameter for executing context-aware algorithms at different levels of healthcare tracking and monitoring applications. Events that occur in an environment can be recognized by identifying relevant actions and also by correlating sequences of actions [11] using fusioning across cross-domains. In [8], Khattak et al. discussed context representation and fusioning as an integral part of any context-aware system. It increases understandability, confidence and reduces redundancy. Contextual Information encompasses basic elements for situation awareness semantically [24]. The obtained contextual information can play substantial roles at different fusion levels by providing convincing clues with contextual spectrum which ranges from sensory data to human-provided information. Alti et al. [10] with their methodological centralized reasoning approach, showed the importance of context recognition and fusion regardless of its source. A three-layered context-aware system infrastructure with inference functionality was proposed in [12], but was unable to prove its deployment for real-world personalized user context identification.

Amongst different context modeling approaches such as: (1) key-value pairs; (2) model-oriented; (3) logic-based; (4) ontologies [25], we are motivated for choosing ontology in context modeling and reasoning. Some of the prominent work using ontologies as a based model is discussed, which includes SOCAM, CoBrA, COSAR, etc. SOCAM [26] is a *Service-oriented Context-Aware Middleware* that works on server-based architecture style with underlying ontology. This provides context-aware services in smart home and vehicle environment. It supports information sensing, semantic representation, and reasoning of context using SOCAM ontology by modeling person, activities along with locations leaving out nutrition contextual aspects. At the same time, CoBrA [27] is also an agent-based centralized middleware architecture connecting context modeling, reasoning, knowledge sharing through context brokers in an intelligent meeting room for places, agents and events only. COSAR [28] recognizes human activities and reasons context-awareness through hybrid reasoning techniques, i.e., ontological/statistical using mobile network services. However, it also focuses only on performed activities and their location in an activity recognition system.

In order to increase the ontological expressiveness and query answering, *Web Ontology Language* (OWL) and *Semantic Web Rule Language* (SWRL) can be integrated to execute knowledge-base system. OWL can be used for problem-solving modeling in terms of ontological development and SWRL may be applied for effective knowledge inference. Using these technologies, Chi et al. [29] constructed a system of chronic kidney disease dietary consultation and proved its effectiveness. In pervasive environments knowledge-driven approaches [30] provide an opportunity to knowledge engineers and domain experts for specifying domain models for capturing and reasoning over recognized activities. Using knowledge driven techniques, ontologies have proven their abilities in describing a service at a certain point of time in the form of abstract context. These abstract contexts assemble and desegregate knowledge relevant to the current situation, the work by Smirnov et al. [31] proposes a classification methodology related to their use in *Decision Support Systems* based on the fusion of context-based information. OWL knowledge-based systems and SWRL can infer new knowledge regarding habit associations of users in a smart home environment [32].

We have adopted context fusioning techniques in context-awareness with the support of the proposed framework called *Multi-level Context-aware Framework* (mlCAF). In order to obtain real-time abstract and richer context in cross-domains, fusioning can be incorporated to infer the abstract contexts in health and wellness domains. Moreover, our proposed framework enables CA platforms to provide better context to adopt behavior and life style.

## 3. Preliminaries on the Mining Minds Platform

*Mining Minds*, an innovative platform based on concepts of the digital health and wellness paradigm, provides personalized healthcare and wellness support. It is mainly the collection of innovative services, tools, and techniques applied collectively to monitor human’s daily life data, generated from heterogeneous sources. To provide innovative services and utilize different tools, the *Mining Minds* platform architecture [33] is divided into five distinct layers: *Data Curation Layer*, *Information Curation Layer* (ICL), *Knowledge Curation Layer*, *Service Curation Layer* and *Supporting Layer* [34]. In a nutshell, *Data Curation Layer* is in charge of acquisition, processing and persisting the asynchronous, real time, multimodal sensory data obtained through heterogeneous sources like SNS, smart phone, Kinect, wearable biomedical devices etc. This processed sensory data is used by *ICL* [35] to model *Low-level Context* (LLC), and infer *High-level Context* (HLC). Health and wellness knowledge is maintained through domain experts or knowledge engineers in *Knowledge Curation Layer*, which contains rules used to determine personal contexts and behavior. *Service Curation Layer* caters users with personalized situation aware recommendations based on determined context and knowledge from Knowledge Curation Layer. Finally *supporting layer* is responsible for advanced analytics, feedback, privacy and security for the *Mining Minds* platform.

*Mining Minds* supports automatic behavior identification through inferring multimodal human context using heterogeneous sensory data. These contexts are identified at two granular layers namely *Low-level Context Awareness* (LLCA) and *High-level Context Awareness* (HLCA). LLCA comprises of physical activities, emotions, and location, which are obtained through machine learning classification techniques. These are applied over sensory data obtained through several devices, such as smart watches, smart phones or Kinect video devices [34]. Also, food items taken during meals are processed through user’s specific tagged meal images, which are collected using the smart phone camera. This study also handles contexts for people with two different chronic diseases, diabetes and hypertension, by capturing blood glucose levels, blood pressure, and helps in determining health conditions with the help of clinical health contexts. This work portrays more comprehensive state of the user in which dietary habits are attached to physical activities and health conditions for better identification of human behavior using sensing modalities in the *Mining Minds* platform.

*Mining Minds* seamlessly supports people’s lifestyles by intelligently fusing human’s daily physical activities and nutrition information along with emotion and location. Behavioral patterns can be derived using diverse HLCs, modeled through inferring over LLCA. ICL layer utilizes the *Mining Minds Context Ontology* (MMCO), which is considered as conceptual backbone for modeling, representing, and inferring HLCs from LLCs. Existing framework [13] lack capability to capture ontological nutrition, health state LLCs and corresponding HLCs. Nutrition is, as important as, physical activities, these are considered most fundamental parts for human health and well-being. Any of unhealthy behavior relating to physical activity or diet may lead to chronic diseases and premature mortality. However, by managing the physical activities patterns and practical dietary risk for chronic diseases can be reduced.

### 3.1. Role of Physical Activities and Nutrition in Diabetes Management

There is a strong relationship between physical activity, dietary habits, and diabetes for people suffering from some chronic disease. Usually, exercises are recommended to all those people who suffer from diabetes as a part of a glycemic control and overall health [15]. In daily life activities, all such individuals are encouraged to decrease the sedentary activities and advised to get involved in healthy activities. These activities are performed by adapting behavior-change strategies, and technology-based strategies which enable people to promote the adoption and maintenance of physical activities. These physical activities help in blood glucose management and overall health maintenance for individuals with diabetes and prediabetes. There is an association between physical activities and the management of glycemia in the individuals having intermediate hyperglycemia or prediabetes [36]. This study provides a strong evidence regarding how an improvement in physical activities can result in lowering diabetes and cardiovascular risks. This lifestyle modification helps individuals in reversion to normoglycemia health conditions. Han et al. [37] proposed a layered healthcare framework for recognizing user’s activities, identifying and providing unhealthy activity patterns to the caregiver. This study claims the possibility of predicting lifestyle diseases associated with an emotional state such as depression; chronic disease like diabetes using sensory data. It is mainly managed by monitoring long-term disease influenced activities such as like frequent eating, frequent drinking, sleeping disorder, less physical activities, etc. Our previous work handled only physical activities, whereas the presented work includes nutrition and chronic disease domains modeled in the ontology. Ontologies model domain-specific concepts and provide reasoning facilities, for this we evolved our previous ontology, only which handled physical activity contexts.

### 3.2. Mining Minds Context Ontology Evolution

In the context modeling world, ontologies outweigh non-semantic models in terms of knowledge sharing, flexibility, reusability, abstraction and validation [17]. Ontologies are, therefore, extensible, expressive, possess decoupling nature, as knowledge and code can co-exist. Additionally and more specifically ontologies enable derivation of new information based on hierarchical structure from existing underlying concepts using class and properties inheritances. Ontologies are evolvable as they provide rich quality and expandable abstract model by including new concepts along with existing ones with handling support of inconsistencies. Besides semantic modeling, ontologies are also supported with the bunch of reasoners, which can be inherently applied for inference and reasoning tasks. Finally, inferring context using ontologies have several implementation benefits in the application development architecture, as these possess resilience and evolving features. These features make ontologies extendable and adaptation of ontology by the architecture itself. Keeping all the above mentioned facts and benefits, we extended our previous ontology by incorporating all associated contextual features for the study under consideration. The open-source *Mining Minds Context Ontology* (MMCO) [38] models daily life contexts, used for human behavior identification which is inevitable for the provision of personalized health and wellness services. A comparative ontological metrics analysis is presented in Table 1, as how ontology evolution has resulted in an increase in ontology metrics, and axioms. Existing work [6] used around 45 classes to model physical activity contexts but in order to add concepts pertaining to nutrition and chronic disease, the classes count raised to 225 along with associated properties and expressivity which is discussed further in this section in detail.

In *Mining Minds*, human context is understood as any information characterizing the physical, nutritional, emotional, health and social situation of a person leading towards the better identification of human behavior. In the development of OWL2 ontologies, *Classes* act as a source of an abstraction mechanism for organizing resources with similar characteristics. Furthermore, *individuals*, also called instances, are members of the OWL Classes and can be related to others using *properties*. In *Mining Minds*, the *subClassOf* constraint is used for designing, assigning classes and individuals for LLC, and HLC. These HLCs are further categorized as *Physical Activity High-level Contexts* (PA-HLCs), *Nutrition High-level Contexts* (N-HLCs) and *Clinical High-level Contexts* (C-HLCs) *classes*. The main concept of MMCO ontology is the *HighLevelContext* class, which has three *subclasses* as *PhysicalActivityContext*, *NutritionContext* and *ClinicalContext* defining the different high-level contexts in the domains of physical activity, nutrition and clinical. The *Location*, *Emotion*, *Activity*, *Food*, *BloodPressure*, *BloodGlucose*, and *WaterIntake* classes have been described to model the different *Low-level Contexts* (LLCs). These classes are related to the class *HighLevelContext*, with the help of object properties *hasLocation*, *hasEmotion*, *hasActivity* [13], *hasFood*, *hasBloodPressure* and *hasBloodGlucose*.

The scalable MMCO comprehensively models contexts at different abstraction levels. PA-HLC are modeled based on the LLCs belonging to the categories *Activity*, *Location*, and *Emotion*. PA-HLC includes contexts for sedentary and active physical activities such as *OfficeWork*, *HouseWork*, *Amusement*, *Gardening*, *Commuting*, *Sleeping*, *Exercising*, and *Inactivity* discussed in detail by Villalonga et al. [6]. The N-HLC are modeled based on LLCs of the category *Food* which is described in a 57 food-item list and further categorized into 10 broader groups. This categorization and major nutrient identifications are performed in accordance with guidance and suggestions provided by *United States Department of Agriculture* (http://www.usda.gov/wps/portal/usda/usdahome) on daily food consumptions. Specifically, the different recognized Foods are categorized using 10 disjoint subclasses of the *Food* class, namely *Grain*, *Meat*, *SeaFood*, *Eggs*, *MilkAndDairyProducts*, *Legumes*, *Nuts*, *Fruits*, *Vegetable*, and *Snacks*. The identified 57 food items are members of these classes, as can be visualized in MMCO [38]. N-HLC models major food nutrient like Carbohydrates, Protein, and Fats which can be determined using LLCs belonging to the category *Food*. The C-HLCs are modeled based on LLCs comprising of 6 LLCs categories for *BloodGlucose*, 5 LLCs for *BloodPressure* and 3 LLCs for *WaterIntake*. Table 2 shows these underlying LLCs and describes the value ranges for each LLC. These LLCs are assigned based on the *BloodGlucose* values obtained through smart glucose meter, *BloodPressure* values captured through smart blood pressure monitor, and accumulated water intake values obtained through smart cup. Finally C-HLC reflects four major health states for diabetic patients, such as normal, moderate, high risk and very high risk health state.

**Figure 2:**
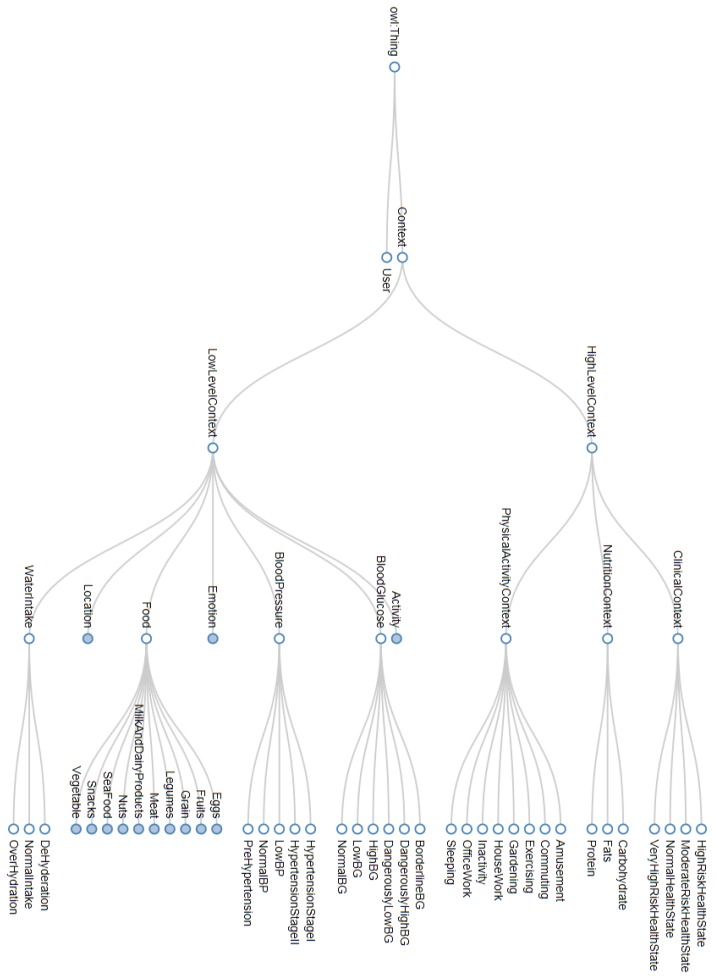
Partial view for extended MMCO: [38]: *LowLevelContext* class and 7 subclasses, *HighLevelContext* class with 3 subclasses, i.e., *PhysicalActivityContext* class with 8 subclasses, *NutritionContext* class with 3 subclasses and *ClinicalContext* class with 4 further subclasses.

**Figure 3:**
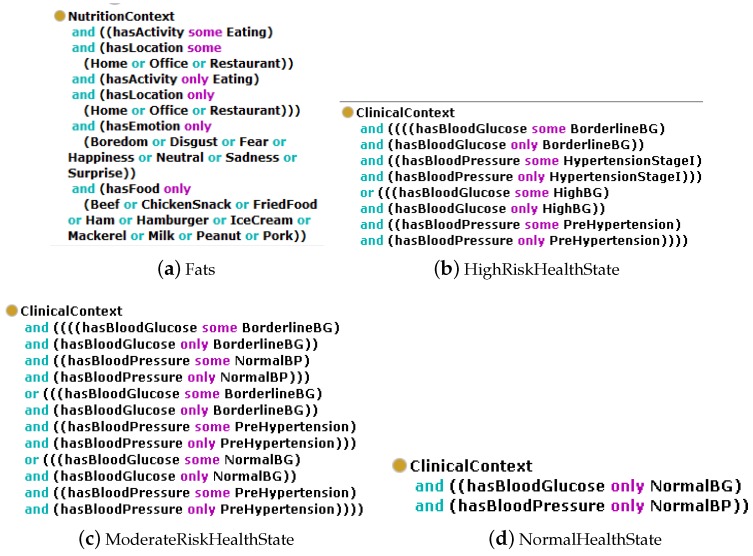
MMCO: Some of example for definitions of the *NutritionContext* and *ClinicalContext* are as (**a**) *Fats*; (**b**) *HighRiskHealthState*; (**c**) *ModerateRiskHealthState*; (**d**) *NormalHealthState*.

The *PhysicalActivityContext* comprises of eight disjoint subclasses as discussed in our previous work [6]. However, the new *NutritionContext* and *ClinicalContext* classes are further divided into disjoint subclasses. The *NutritionContext* class, which represent N-HLCs has three disjoint subclasses decided based on major nutrient in food items, they are named as *Carbohydrates*, *Protein*, and *Fats*. The *ClinicalContext* class, which represents C-HLC class, is further subdivided as *NormalHealthState*, *ModerateHealthState*, *HighRiskHealthState*, and *VeryHighRiskHealthState* also shown in Figure 2 as a partial part of MMCO. Each *NutritionContext* and *ClinicalContext* subclasses are defined through complement classes, existential and universal axioms. These axioms constitute the necessary conditions required for equivalent anonymous class definitions. The equivalent anonymous classes for these *NutritionContext* and *ClinicalContext* subclasses have been described in Protégé and are shown in Figure 3.

In order to explain equivalent Class concepts, the N-HLC is modeled via the *Fats* class, Figure 3a, is considered as being equivalent to the anonymous class with certain constraints like *NutritionContext and (hasActivity some Eating) and (hasLocation some (Home or Office or Restaurant)) and (hasActivity only (eating) and (hasLocation only (Home or Officeor Restaurant)) and (hasEmotion only (Happiness or Disgustor Boredomor Anger or Neutral)) and (hasFood only (Beef or Chicken Snack or Fried Food or Ham or HamBurger or IceCream or Mackerel or Milk or Peanut or Pork))*. This assures that to become instance of the defined class *Fats*, an individual of the *NutritionContext* class must have an object property of type *hasActivity*, which has to be associated with an instance of the *Eating* class, and this object property must take as only value an instance of the *Eating* class. Moreover, the instance of the *NutritionContext* class must also meet the condition for having a property of type *hasLocation* relating to an instance of the *Home or Office or Restaurant* class and only to an instance of the *Home or Office or Restaurant* class. The instance referring the *NutritionContext* class has an object property of type *hasEmotion*, this property must also relate to an instance of the *Happiness* class, the *Disgust* class, the *Boredom* class, the *Anger* class, or the *Neutral* class. Finally, the property of type *hasFood*, must relate to an instance of the *Beef* class, the *ChickenSnack* class, the *FriedFood* class, the *Ham* class, the *HamBurger* class, the *IceCream* class, the *Mackerel* class, the *Milk* class, the *Peanut* class, or the *Pork* class. In the definition and for inferring of *Fats* class the *hasFood* object property and *hasActivity some Eating* axioms are mandatory due to existential and universal restrictions on the involved object properties in addition to *hasLocation* and *hasEmotion* object properties. Similar class concept equivalence definitions are formulated for other N-HLCs, *Carbohydrates* and *Protein* classes and C-HLC subclasses i.e., *NormalHealthState*, *ModerateHealthState*, *HighRiskHealthState*, and *VeryHighRiskHealthState*.

### 3.3. High-Level Context Awareness in a Nutshell

**Figure 4:**
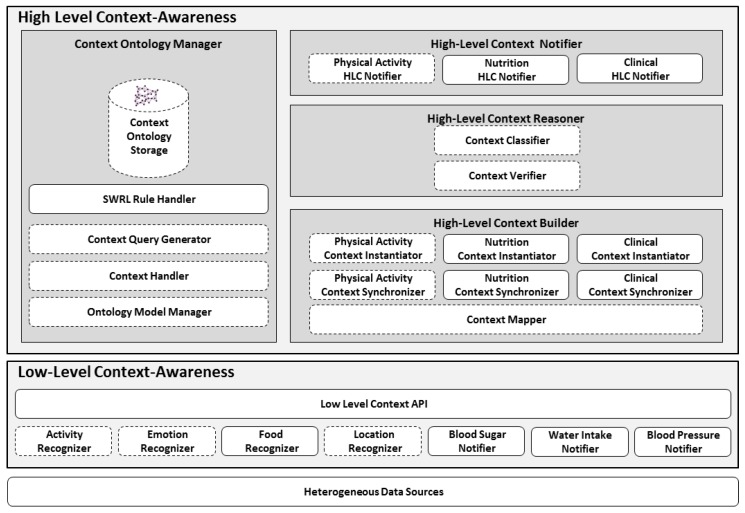
Mining Minds High-Level Context Awareness extended architecture.

In *Mining Minds*, the core technologies devised for the inference and modeling of the user’s context constitutes the *Information Curation Layer*, which is further subdivided to *Low-level Context Awareness* (LLCA) and *High-Level Context Awareness* (HLCA) as mentioned in Figure 4. The sensory data obtained through user is converted to *Low-level Context* (LLC) using machine learning approaches and it also becomes the basis for inferring in order to obtain *High-Level Contexts* (HLC) of three types i.e., *Physical Activity High-Level Context* (PA-HLC), *Nutrition High-Level Context* (N-HLC) and *Clinical High-Level Context* (C-HLC). The components drawn in by bold lines are the architectural perspective contributions to the existing work in order to improve context recognition and better behavioral modeling using nutritional and clinical information. LLCs contain seven main context categories *Activities*, *Locations*, *Emotions*, *Food*, *BloodSugar*, *BloodPressure*, and *WaterIntake*, which are recognized through respective recognizers. *Activity* LLC is identified from the body actions and mobility, positioning systems helps in identifying *Location* LLC, *Emotion* LLC is obtained from the user sentiments detected through voice recognizing systems [35]. *Food* LLC is obtained using labels of food items sent by users by capturing pictures of meals by using smart-phone. *BloodSugar* values are taken using a smart glucose meter, which communicates with the *Mining Minds* platform using API, *BloodPressure* is measured using the smart blood pressure monitor, and *WaterIntake* notifier gets input from the smart cup.

HLCA as shown in Figure 4 consists of four main components: *High-Level Context Builder*, *High-Level Context Reasoner*, *High-Level Context Notifier*, and *Context Ontology Manager*. The HLC Builder receives the low-level information activities, emotions, locations, food, BloodSugar, BloodPressure and WaterIntake levels identified by LLCA and maps into ontological concepts named as unclassified context. The *Physical Activity Context Synchronizer* searches for concurrent LLC information related to activities, emotions, and locations whereas the *Nutrition Context Synchronizer* takes Food LLC into account additionally. Similarly, *Clinical Context Synchronizer* collects BloodGlucose and BloodSugar LLCs and synchronize them. The *Physical Activity, Nutrition and Clinical Context Instantiator* create a new instance of an *unclassified HLC* which links to the corresponding LLCs w.r.t. time. The unclassified PA-HLC, N-HLC and C-HLC are communicated to the *HLC Reasoner* for its validity and classification. The *Context Verifier* examines the syntactic and semantic consistency of the unclassified context. The *Context Classifier* administers ontological inference and classifies the unclassified context into one of the different PA-HLC, N-HLC or C-HLC. The *PA-HLC, N-HLC and C-HLC Notifier* notifies the newly identified PA-HLC, N-HLC and C-HLC to other *Mining Minds* layers for the creation of personalized health, wellness services [34] and necessary recommendations. The fourth component *Context Ontology Manager* is responsible for the persistence of MMCO, storage of LLC, PA-HLC, N-HLC, C-HLC and supports SPARQL/SQWRL queries for retrieval of LLCs, HLCs and behavioral contexts.

## 4. Context Reasoning

The main purpose of context reasoning is to check the consistency of contexts as well as deducing high-level implicit context information from low-level explicit contexts. In this study, ontology-based reasoning is performed with additional assistance for user-defined SWRL rules and SPARQL/SQWRL queries.

### 4.1. Ontology Based Reasoning

In Ontology-based reasoning, inferring is done based on ontological constraint-based rules exploiting *transitive property*, *subClassOf*, *subPropertyOf*, *disjointWith* and *inverseOf* properties. However, inclusion of complex rules increases the expressibility of ontological model, which enhances the ability of handling the multi-level context assertions.

### 4.2. SWRL Based Reasoning/SQWRL Based Retrieval

SWRL rules, an extension to OWL-DL is one of semantic rule representation for representing knowledge. These rules are designed to obtain the desired outcome based on already stored facts meeting several conditions and adds more power to OWL capabilities for deductive reasoning. Integration of SWRL to ontological based inferring systems have shown their importance for context-aware applications [41]. Ontological models have limitations for handling complex rules as in our case fusing multi-level cross-domain contexts, which are discussed in subsequent sections. SWRL adds flexibility in fusing Multilevel contexts thus resulting in inferring of additional contexts using already asserted facts. SQL like querying is human understandable, an introduction of *Semantic Query-Enhanced Web Rule Language* (SQWRL) on top of SWRL provides SQL-like operators for querying and extracting semantic information. SQWRL uses SQWRL Query API (https://github.com/protegeproject/swrlapi/wiki/SQWRLQueryAPI) interface for executing the queries and accessing the results in a two-dimensional table.

## 5. Multi-Level Cross-Domain Context Fusioning

In the *Mining Minds*, we ideally focused on a solution catering contexts from multiple domains, as a proof of concept, we considered PA-HLC, N-HLC and C-HLC such that these are handled separately on one-level, fused together on another level and finally more concrete and abstract multi-level cross-domain contexts are obtained suggesting human behavior and lifestyle. This has helped in increasing the ability to apply our proposed solution to multi-domains, which initially was only composed of physical activity domain.

**Figure 5:**
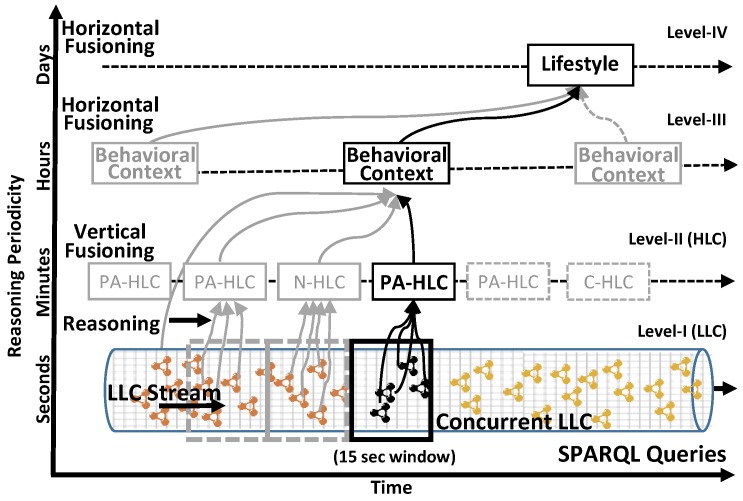
Exemplification of representation for mlCAF at different abstraction levels using Vertical and Horizontal Fusioning.

In Figure 5, a summarized overview is provided how the overall mechanism of mlCAF utilizing vertical fusioning and horizontal fusioning are performed at different levels, particularly with time constraints and reasoning periodicity. This horizontal fusioning is achieved using SWRLAPI (https://github.com/protegeproject/swrlapi) which works on top of OWL-based SWRL and SQWRL [42] query language discussed in the implementation Section 6. SQWRL uses SWRL’s conjunctive semantic structure as its formal underpinning by providing novel set operators used to perform closure operations such as negation, additionally with counting, and aggregation.

### 5.1. Vertical Fusioning: Context Inferring in the Mining Minds

**Figure 6:**
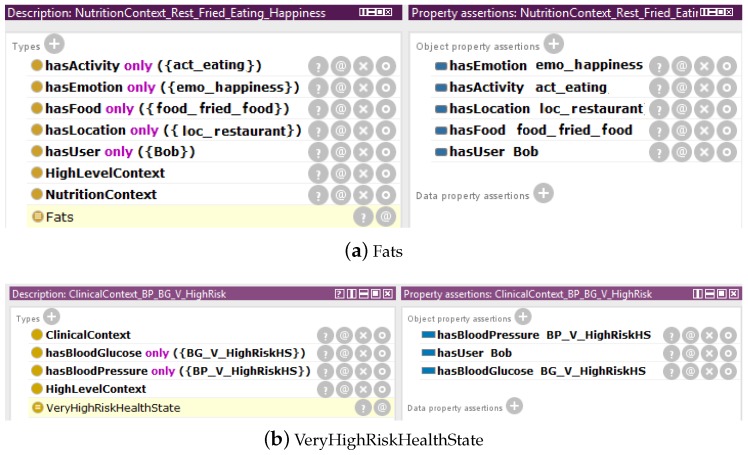
Instances of the *NutritionContext* and *ClinicalContext* classes which are classified as being members of the defined *NutritionContext* and *ClinicalContext* subclasses using the Pellet reasoner in Protégé. The inferred classes are highlighted in yellow: (**a**) *Fats* and (**b**) *VeryHighRiskHealthState* are inferred.

The MMCO is utilized to infer PA-HLC, N-HLC and C-HLC in a vertical fusioning manner utilizing ontological reasoning (Level-II). Using Pellet reasoner, an instance of the *PhysicalActivityContext* class, i.e., an unclassified PA-HLC, can be determined to be associated with one of the eight *PhysicalActivityContext* subclasses as mentioned in [13]. An instance of the *NutritionContext* class can be determined to be a member of one of the three *NutritionContext* subclasses: *Fats*, *Carbohydrates*, and *Protein*. Similarly, an instance of the *ClinicalContext* class, can be determined to be a member of one of the four *ClinicalContext* subclasses: *NormalHealthState*, *ModerateHealthState*, *HighRiskHealthState*, and *VeryHighRiskHealthState*. The instances of the *Food* class are asserted through the *hasFood* property, whereas instances of the *BloodGlucose* and *BloodPressure* classes are asserted using *hasBloodGlucose* and *BloodPressure* properties respectively. Reasoning in OWL is based on the *Open World Assumption* (OWA), which means that an assumption cannot be made for something if it does not exist until its nonexistence is declared explicitly. Following is an example discussed in order to illustrate the modeling principles and the inference logic through MMCO in the Protégé tool as shown in Figure 6. Figure 6a shows an instance of the *NutritionContext* class for which the object property *hasActivity* has been asserted to take the value *act_eating*, *hasEmotion* object property has been asserted to take the value *emo_happiness*, *hasFood* property has been asserted to take the value *food_fried_food*, *hasLocation* property has been asserted to take the value *loc_restaurant* and *hasUser* property has been asserted to take the value *Bob*; where *act_eating* is an instance of the *Eating* class, *emo_happiness* is an instance of the *Happiness* class *food_fried_food* is an instance of the *Fried_Food* class and *loc_restaurant* is an instance of the *Restaurant* class. Due to the OWA, the instance of the *NutritionContext* class has been asserted the type *(hasActivity only act_eating)* and the type *(hasEmotion only emo_happiness)* and *(hasFood only food_fried_food)* and the type *(hasLocation only loc_restaurant)* and the type *(hasUser only Bob)*. The reasoner is used to automatically classify this instance of the *NutritionContext* class. The instance complies with the *Fats* class definition; therefore, it is classified as being a member of the *Fats* class. The *Context Reasoner* infers *Fats* using vertical fusioning as LLC belongs to nutrition domain i.e., *Food* name and *Activity Eating* comes from physical activity domain as shown in Figure 6a. Similarly, vertical fusioning infers *Carbohydrates* and *Protein*. These contexts are concurrent, due to their overlapping temporal properties, we considered them as they are handled through vertical fusioning inferring. This process is scalable as we can fuse as many concurrent contexts depending on ontological modeling and service requirements.

**Figure 7:**
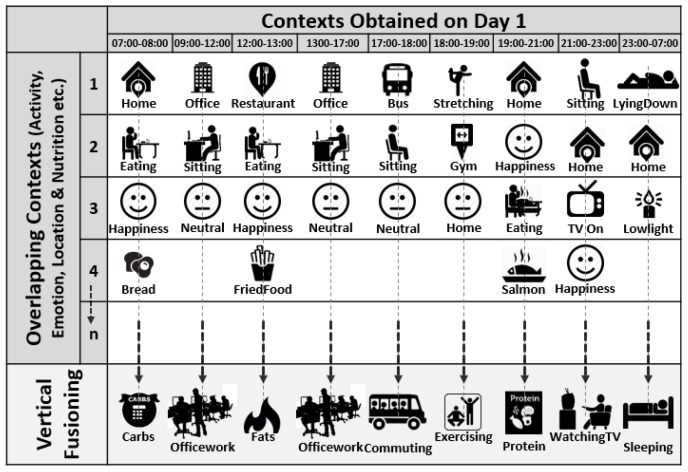
Example scenario explaining Vertical Fusioning using overlapping Low-level contexts.

An example scenario is plotted in Figure 7 explaining how vertical fusioning works on concurrent contexts i.e., Level-II fusioning as discussed previously in Section 5.1. These overlapping contexts are vertically fused to obtain abstract context form, using ontological based reasoning. Different vertically fused contexts are plotted based on overlapping low-level Activity, Emotion, Location and Food contexts.

### 5.2. Horizontal Fusioning: Context Inferencing in the Mining Minds

The SWRL rules have been designed over and above with MMCO constraint-based rules, by keeping temporal characteristics of concurrent low-level contexts, inferred PA-HLC and N-HLC in view. SWRL combines these atomic dynamic contexts to provide a complete picture of highly interrelated atomic low-level contexts, PA-HLC and N-HLC. The general form of SWRL rule looks like as mentioned below.

(1) $$\alpha_{1}\wedge \alpha_{2}\wedge \alpha_{3}\ldots \ldots \wedge \alpha_{n}->\beta_{1}\wedge \beta_{2}\ldots \ldots ..\wedge \beta_{m}$$

where $\alpha_{i}$ symbolizes atomic low-level contexts (i.e., Activity, Location, Emotion, Food and clinical contexts), PA-HLCs, N-HLCs, C-HLCs and properties connecting these contexts whereas $\beta_{i}$ represents outcomes of $\alpha_{i}$ in abstracted way named as behavioral contexts. These $\alpha_{i}$, $\beta_{i}$ can be of the form C(x), P(x,y), etc., where C represents OWL concept description and P represents OWL property, and x, y are termed as Datalog variables. On both sides of arrows, these are connected using conjunctions. The resulting behavioral contexts are then utilized for lifestyle identification.

**Figure 8:**
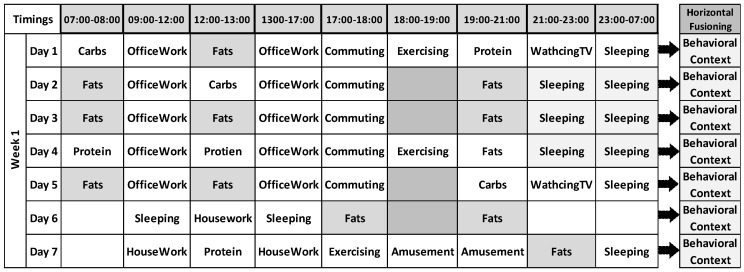
Illustration scenario explaining Horizontal Fusioning.

The contexts obtained using vertical fusioning do not provide enough evidence to establish human behavior as long as they belong to user’s context at the point of time as shown in the example scenario, Figure 8. In order to get better picture, a mechanism is devised for fusing vertically fused contexts to a more abstract form, such that it can result into better behavior modeling. In this case, the time window will appear bigger rather than in seconds as in the case of VF. We obtained higher abstraction (Level-III, see Figure 5) for contexts spanning even more than a day, by fusing several vertically fused contexts using defined SWRL/SQWRL rules and queries. In order to prove the HF concept, considering these inferred PA-HLC, N-HLC and C-HLC along the time-line, they are reasoned at different point of time, means horizontally along with the timeline. With the introduction of SWRL/SQWRL rules additionally, as mentioned in Table 3 more multi-level contexts are obtained explained in next sections.

Using SWRL rules, retrieval through SQWRL, horizontal fusioning of cross-domain contexts from different levels is accomplished.

## 6. Implementation Details

The following sections discuss in detail about the implementation test environment, which was constructed using *Semantic Web APIs*. Furthermore the evaluation for results and introduction of advanced queries along with detailed description are presented.

### 6.1. Test Environment

All experiments were performed on a single machine under 64bit Enterprise edition of *Microsoft Windows 7* on the top of AMD A10-5800K APU with 12 GB of RAM. Java engine was executed with Eclipse IDE Luna that ran using JRE 1.8.0_45-b15 64-bit version.

### 6.2. Semantic Web APIs Usage Details

The implementation [14] of HLCA in mlCAF is performed in Maven (https://maven.apache.org/) based project management with Java along with available open source library, Apache Jena v2.11.2 (https://jena.apache.org/). The component *High-Level Context Builder* receives unstructured low-level information, namely locations, activities, emotions, food, BloodGlucose, and BloodPressure. The *Context Mapper* maps received low-level information in the form of the label for *Activity*, *Location*, *Emotions*, *Food*, *BloodGlucose*, and *BloodPressure* along with meta-data comprising of Start-time, user-info, etc., using semantic web framework including RDFS [43], OWL2 [44], and OWL-API (http://owlapi.sourceforge.net/).

**Figure 9:**
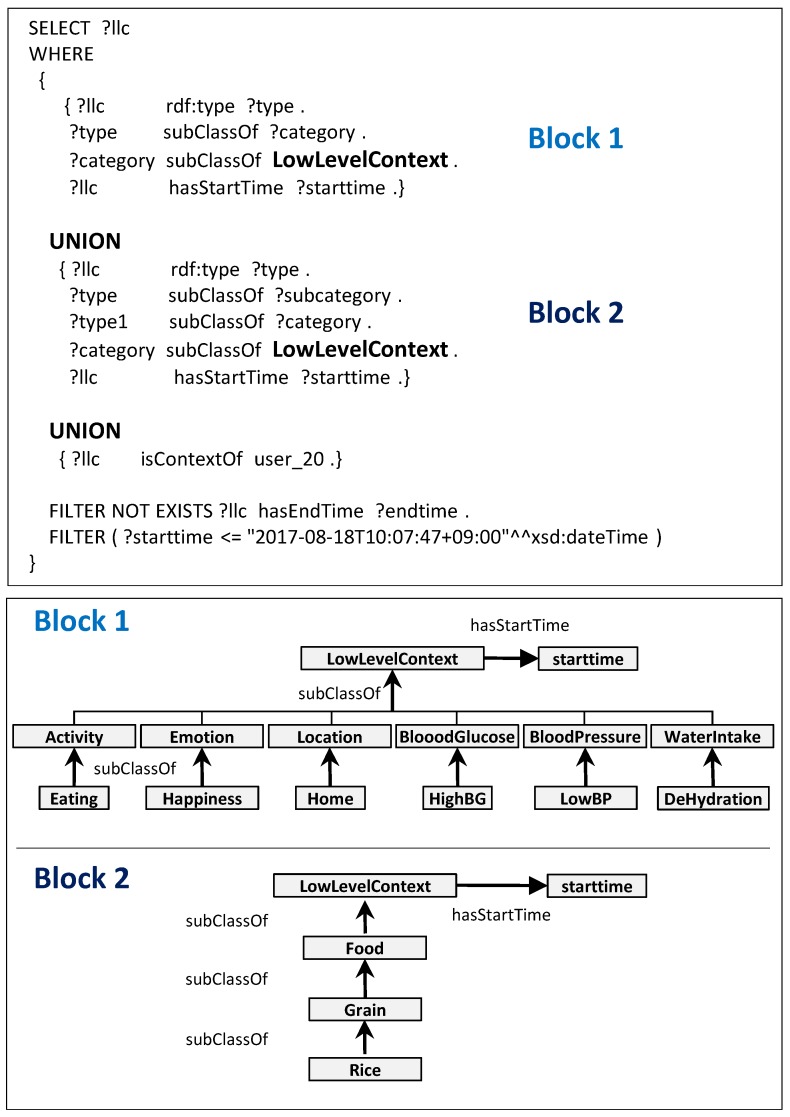
Evidential mappings of MMCO with SPARQL Query while retrieving Concurrent low-level Context w.r.t timestamps.

**Figure 10:**
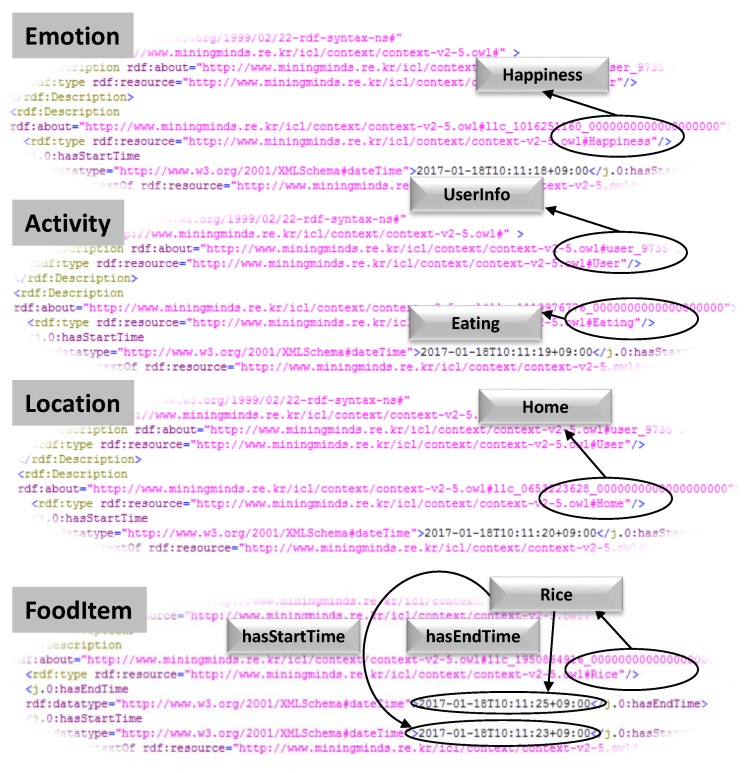
Context Synchronizer: Synchronizing low-level contexts w.r.t timestamps.

**Figure 11:**
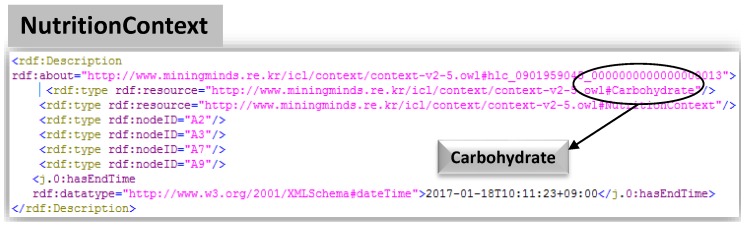
Inferred NutritionContext: Carbohydrate (Major Nutrient) in *Rice* Fooditem LLC.

It transforms this information into ontological triple format. The *Physical Activity Context Synchronizer* takes concurrent low-Level contexts like *Activity*, *Location* and *Emotion* valid at the same moment in time. Similarly, *Nutrition Context Synchronizer* processes whenever food low-level context has arrived. *Nutrition context synchronizer* takes the “Eating” activity low-level context along with Food low-level context in addition to emotion and location. The synchronized LLCs are obtained by SPARQL queries [45] using *Union* operators capable of combining two or more SELECT statements as mentioned in Figure 9 labeled as *Block 1 and Block 2*. *Block 1* retrieves results pertaining to underlying cross-domain LLCs whereas *Block 2* structure is designed to retrieve nutrition LLCs. The obtained synchronized LLCs are remodeled to *unclassified HLCs* depending on the concurrent LLCs. These different synchronized LLCs can be visualized in Figure 10 coupled w.r.t their timestamps in RDF format. The *High-Level Context Reasoner* receives either three types of *unclassified HLCs*, i.e., PA-HLC, N-HLC or C-HLC for their verification and classification. Firstly, the *Context Verifier* checks the semantic and syntactic consistency of the *unclassified HLCs* and then the *Context Classifier* performs the ontological inference and identifies the membership of the *unclassified HLCs* with the help of Pellet (https://www.w3.org/2001/sw/wiki/Pellet), an open source OWL-DL reasoner. All the contexts are stored in Jena TDB (https://jena.apache.org/documentation/tdb/). The *Physical Activity Context Notifier* or *Nutrition Context Notifier* makes the newly inferred HLCs Figure 11 to the *Mining Minds* or any third party entity for behavior analysis and recommendation generation.

### 6.3. Experimental Results

**Figure 12:**
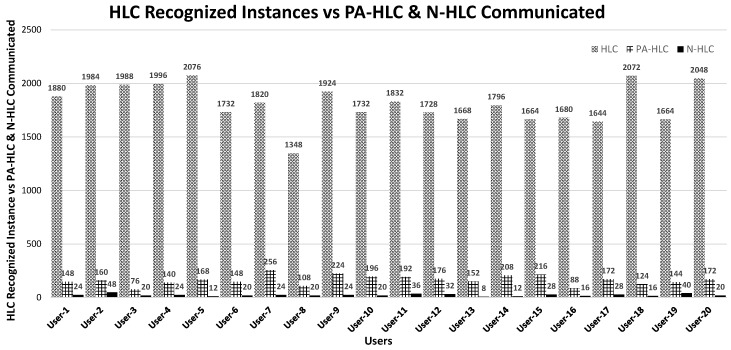
HLC Recognized Instances vs PA-HLC and N-HLC Communicated.

The evaluation has consisted in the collection of around 141,872 *Low-Level Contexts* and 36,276 recognized *High-Level Contexts* belonging to 20 different adult volunteers in real environment using MM platform involving mlCAF framework. In example scenario, we considered user’s LLC, PA-HLC and N-HLC spanning over-a-week as a case study to explain and provide enough evidence for vertical fusioning and horizontal fusioning, so that we can validate sedentary and active behaviors. The graphical representation mentioned in Figure 12 shows the HLCs inferred for different users with vertically fused PA-HLC and N-HLC. This representation gives intuition how vertical fusion helps in reducing huge volume of low-level contexts to PA-HLC and N-HLC. So instead of communicating all HLCs with third party entities, only whenever the change occurs, the respective vertically fused HLCs are delivered thus reducing network communication amongst entities. Out of 36,276 HLCs, only 4144 PA-HLC, N-HLC, and C-HLC were communicated behavior modeling APIs as shown in Figure 12.

**Figure 13:**
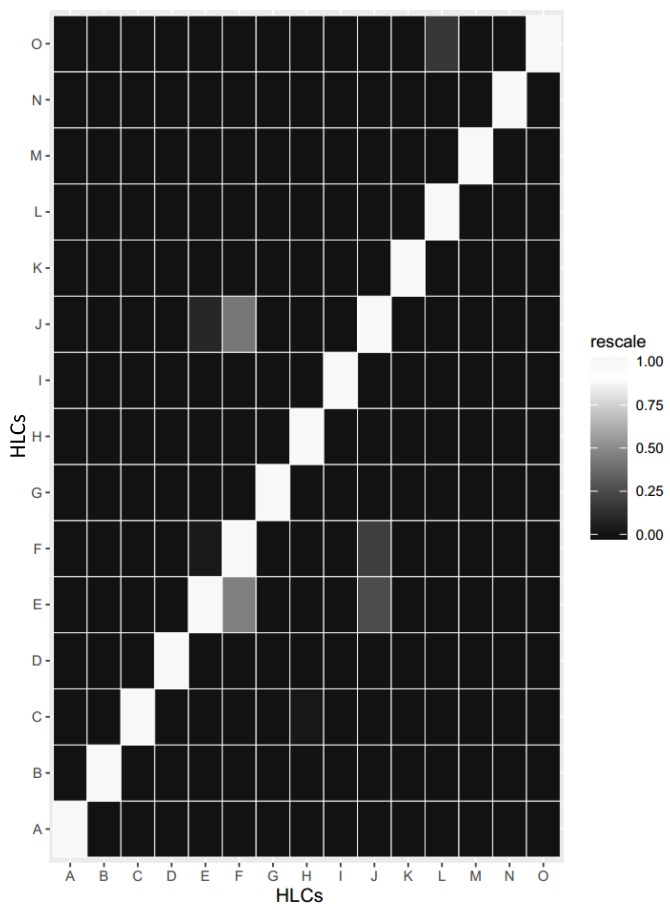
Confusion Matrix expressed as heat map for recognized HLCs (PA-HLC, N-HLC, and C-HLC). Legend: A = Amusement, B = Commuting, C = Exercising, D = Gardening, E = Carbohydrate, F = Fats, G = Housework, H = Inactivity, I = OfficeWork, J = Protein, K = Sleeping, L = HighRiskHealthState, M = ModerateRiskHealthState, N = NormalHealthState, O = VeryHighRiskHealthState.

**Figure 14:**
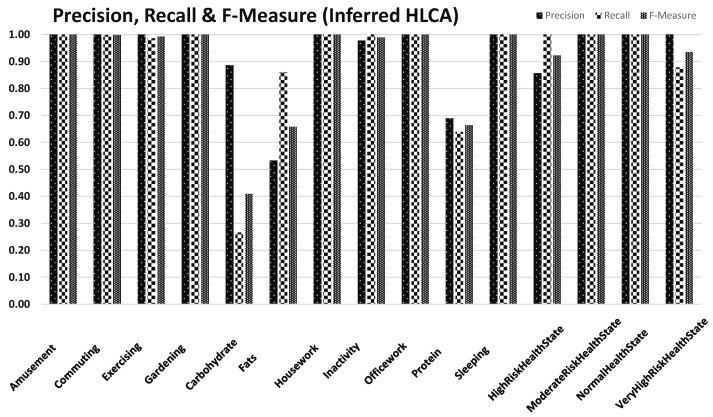
Precision, Recall and F-Measure for recognized PA-HLCs, N-HLCs and C-HLCs.

Heatmap confusion matrix representation shown in Figure 13 provides accuracies for recognized HLCs (i.e., PA-HLCs, N-HLCs and C-HLCs). Around 7.35% HLCs were not classified by the reasoner whereas 92.65% HLCs were realized correctly. It is evident that good accuracy results were achieved using cross-domain context fusioning, however, some missing LLCs lead to the declined accuracies for HLCs especially for N-HLCs. For a collected dataset, we evaluated how HLCA correctly inferred high-level contexts based on low-level contexts for the 15-s sliding window. We used the precision and recall measures to evaluate HLCA in mlCAF as shown in Figure 14 to address our performance challenges. The high inferring accuracy proved that HLCs are rarely misclassified but the problem arises when missing LLC leads to wrongly recognized HLC.

- Precision: Number of HLC correctly inferred by mlCAF divided by the total number of HLC defined in MMCO.
- Recall: Number of HLC correctly answered by the mlCAF divided by the total number of mlCAF in the dataset.

**Figure 15:**
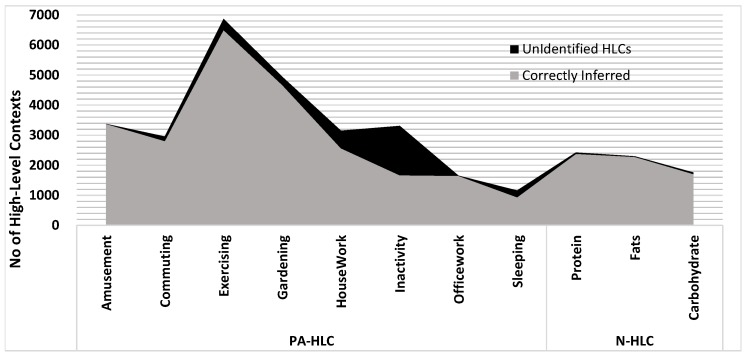
Unidentified PA-HLCs, and N-HLCs due to missing LLCs.

Uncertainty exists in ontological reasoning which leads to ambiguous results. By carefully analyzing *Unidentified PA-HLC* and *N-HLC*, it was realized that *Unclassified HLCs* are inferred to *Unidentified PA-HLC* and *N-HLC* with some missing or wrongly identified low-level contexts as shown in Figure 15.

**Figure 16:**
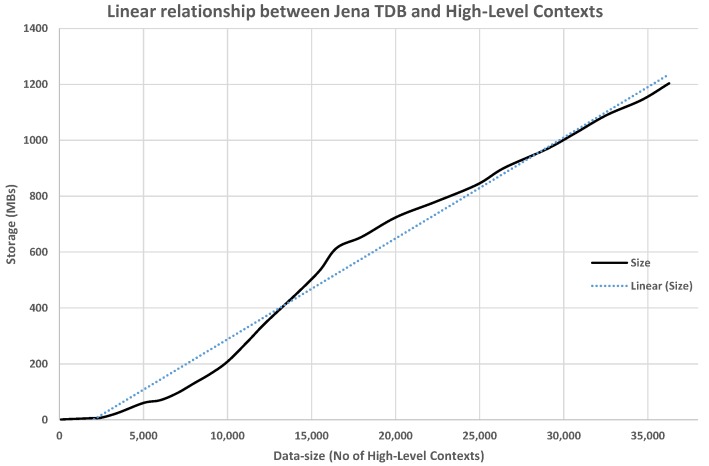
Impact of No of instances on Jena TDB.

In order to check disk space utilization, resources were examined by persisting RDF dataset pertaining to 20 different users. Figure 16 shows a linear relationship between Jena TDB resource utilization and increasing number of contexts.

### 6.4. SQWRL based advanced Queries

In context-awareness for user personalization, SWRL/SQWRL queries were designed to aid horizontal fusioning to obtain human behavior. As mentioned in Table 3 the antecedent of each SWRL/SQWRL represents a conjunction (or union) of the user, LLC, PA-HLC, N-HLC and associated properties. These rules are constructed as per logic flow described in Figure 5. Daily physical activities, food intake and eating patterns can also have substantial effects on human health. Food intake frequency can induce healthy benefits and influences of the timing of meals and frequency on health may be large [46]. Healthy eating can be judged by identifying meal frequency and has a deep relationship with physical activities. In order to observe nutrition intake quantity *meal frequency* is also determined and queried. In this study, we presented SQWRL related to query constructions for behavior context based on user sedentary behaviors and also considered identification of meal frequency.

**Rule 1. Sedentary Behavior**

The meaning of the rule is as follows:

(i) If the user “u” has location “Loc_Gym”.
(ii) The user “u” has PA-HLC as “Exercising” and it has some start time and end time.
(iii) The duration is less than two hours.
(iv) The difference in a number of days is less than seven days i.e., in a week.
(v) User “u” has “Sedentary Behavior” if “Exercising” PA-HLC is less than two hours in a week.

**Rule 2. Lightly Active**

(i) If the user “u” has a low intensity activity such as walking “Act_Walking” which is *subClassOf* of PA-HLC.
(ii) The user “u” has PA-HLC as “Exercising” with start-time and end-time.
(iii) If the duration is between one hour and three hours.
(iv) If the difference in the number of days is less than seven days i.e., in a week.
(v) This SQWRL query selects the users “u” a “Lightly Active” if “Exercising” PA-HLC is between one hour and three hours but with LLC *Activity* as *Act_Walking* in a week.

**Rule 3. Moderately Active**

(i) If the user “u” has a moderately intensive activity such as Running with instance “Act_Running”, which is *subClassOf* of PA-HLC.
(ii) The user “u” has PA-HLC as “Exercising” with start-time and end-time.
(iii) If the duration is between three hour and five hours.
(iv) If the difference in the number of days is less than seven days i.e., in a week.
(v) This SQWRL query selects the users “u” a “Moderately Active” if “Exercising” PA-HLC is between three hours and five hours but with LLC *Activity* as *Act_Running* in a week.

**Rule 4. Very Active**

(i) If the user “u” has high intensive “Exercising” with start-time and end-time as PA-HLC.
(ii) The duration of “Exercising” is between one hour and three hours.
(iii) The intensive “Exercising” is being performed daily.
(iv) This designed SQWRL query will retrieve a list of all users who perform high intensive “Exercising” and they are termed as having *Very Active* behavioral context as they are regularly engaged in performing physical activities.

**Rule 5. Extremely Active**

(i) If the user “u” has vigorous-intensive “Exercising” with start-time and end-time as PA-HLC.
(ii) The duration of “Exercising” is between one hour and three hours.
(iii) The vigorous-intensive “Exercising” is being performed twice a day.
(iv) This SQWRL query will retrieve a list of all users who perform vigorous-intensive “Exercising” and the resultant users are considered to be of *Extremely Active* behavior, who perform intensive workouts twice a day having an overall duration of two hours or more.

**Rule 6. Meal Frequency**

(i) If the user “u” has some activity “Eating”.
(ii) The user “u” has N-HLC as “Eating” and it has some start time and end time.
(iii) Count the activity “Eating” per day.
(iv) The presented SQWRL query calculates the frequency of an activity “Eating” in a day for the users and returns all users with given conjunctive conditions.

These captured human behaviors using VF and HF are transmitted to other Mining Minds platform layers for further support to user’s health and wellbeing by monitoring physical activity, nutrition, and health-related states.

## 7. Conclusions and Future Work

This paper has presented the ontology-based modeling and cross-domain context fusioning, associated implementation using *multi-level Context-aware Framework* mlCAF, methods for transforming *Low-Level Contexts* (LLCs) into *Physical Activity High-Level Context* (PA-HLC), *Nutrition High-Level Context* (N-HLC) and *Clinical High-Level Context* (C-HLC). These PA-HLC, N-HLC and C-HLC are transformed using ontological based inference resulting in the vertical fusioning for cross-domain low-level contexts. In this work, the *Mining Minds Context Ontology* (MMCO) was extended to cater cross-domain contexts from physical activity, nutrition and clinical domains in order to identify major nutritional patterns and behaviors for users. It is pertinent to mention that a broader picture of human behavior modeling and lifestyle determination is obtained by using SWRL/SQWRL additionally as a horizontal fusioning, which keeps on creating associations amongst PA-HLC, N-HLC, C-HLC, and even LLCs at different levels. The main motivation behind this work lies in correctly identifying user’s daily life contexts, behavior identification, as lifestyle, which lead to better community care and wellness. So the key direction that underpins our ongoing research in the *Mining Minds* involves real-time human behavior modeling and lifestyle prediction using cross-domain contexts. In this regard, experimental results indicate a context recognition average accuracy of around 92.65% for the inferred cross-domain contexts at different fusioning levels. Moreover, investigations are still underway for the identification for more efficient context fusioning mechanism through semantic technologies. it is confirmed that there is still a need for relevant data within in the referred time, which is to be used in the *Mining Minds* with MMCO for more accurate PA-HLC, N-HLC and C-HLC contexts situation analysis. In addition, creation and execution of more SWRL/SQWRL rules will have a significant impact on the overall platform in terms of better behavioral modeling and lifestyle identification. In future, we are planning to extend SWRL/SQWRL for lifestyle modeling and perform prediction analysis using inductive reasoning by applying data-driven approaches on gathered dataset i.e., A-Box assertions in the Jena Triple-store Database. 

## Figures and Tables

**Table 1:** Ontology Metrics during Ontology Evolution.

MMCO Metrics	Metrics Detail	MMCO V2.0 (Existing Work) [6]	MMCO V3.0 (Extended Work)
Metrics	Axioms	793	1092
	Logical Axioms	624	859
	Class count	45	225
	Object Property count	3	25
	Individual count	114	157
	DL Expressivity	ALCO	ALCHOF(D)
	Asserted Triples	0	3312
	Inferred Triples	0	6041
Class Axioms	SubClassOf	32	222
	Equivalent Classes	9	17
	Disjoint Classes	5	14
Individual Axioms	Class Assertion	360	58
	Object Property Assertion	212	27
	Data Property Assertion	0	1

**Table 2:** Low-level Context labels assigned based on ranges of values.

Category	Low-Level Context Labels	Value Ranges
Blood Glucose [39] (mg/dL)	DangerouslyHighBG	≥315
	HighBG	>215 & <280
	BorderlineBG	>120 & <180
	NormalBG	>70 & <108
	LowBG	>50 & <70
	DangerouslyLowBG	≤50
Blood Pressure (Systolic/Diastolic) [40]	HypertensionStageII	≥160/100
	HypertensionStageI	>140/90 & <159/99
	PreHypertension	>120/80 & <139/89
	NormalBP	≤120/80
	LowBP	<90/60
Water Intake (mL)	OverHydration	>2000 mL
	NormalIntake	approx. 2000 mL
	Dehydration	<2000 mL

**Table 3:** SWRL/SQWRL definitions: PA-HLC, N-HLC and LLC involved in Horizontal Fusioning for Behavior modeling.

Rule	Behavioral Contexts	SWRL/SQWRL Horizontal Fusion Rules
1	Sedentary Behavior	User(?u) ∧ hasLocation(?u, Loc_Gym) ∧ isContextOf(?u, ?PAC-HLC) ∧ swrlb:equal(?PAC-HLC, “Exercising”) ∧ hasStartTime(?PAC-HLC, ?starttime) ∧ hasEndTime(?PAC-HLC, ?endtime) ∧ temporal:duration(?h, ?starttime, ?endtime, ”Hours”) ∧ temporal:duration(?d, ?starttime, ?endtime, “Days”) ∧ swrlb:lessThan (?h, 2) ∧ swrlb:lessThan(?d, 7) -> sqwrl:select(?u, ?h, ?d)
2	Lightly Active	User(?u) ∧ hasActivity(?u, Act_Walking) ∧ isContextOf(?u, ?PAC-HLC) ∧ swrlb:equal(?PAC-HLC, “Exercising”) ∧ hasStartTime(?PAC-HLC, ?starttime) ∧hasEndTime(?PAC-HLC, ?endtime) ∧ temporal:duration(?h, ?starttime, ?endtime, “Hours”) ∧ temporal:duration(?d, ?starttime, ?endtime, “Days”) ∧ swrlb:greaterThan(?h, 1) ∧ swrlb:lessThan(?h, 3) ∧ swrlb:equal(?d, 7) -> sqwrl:select(?u, ?h, ?d)
3	Moderately Active	User(?u) ∧ hasActivity(?u, Act_Running) ∧ isContextOf(?u, ?PAC-HLC) ∧ swrlb:equal(?PAC-HLC, “Exercising”) ∧ hasStartTime(?PAC-HLC, ?starttime) ∧ hasEndTime(?PAC-HLC, ?endtime) ∧ temporal:duration(?h, ?starttime, ?endtime, “Hours”) ∧ temporal:duration(?d, ?starttime, ?endtime, “Days”) ∧ swrlb:greaterThan(?h, 3) ∧ swrlb:lessThan(?h, 5) ∧ swrlb:equal(?d, 7) -> sqwrl:select(?u, ?h, ?d)
4	Very Active	User(?u) ∧ isContextOf(?u, ?PAC-HLC) ∧ swrlb:equal(?PAC-HLC, “Exercising”) ∧ hasStartTime(?PAC-HLC, ?starttime) ∧ hasEndTime(?PAC-HLC, ?endtime) ∧ temporal:duration(?h, ?starttime, ?endtime, “Hours”) ∧ temporal:duration(?d, ?starttime, ?endtime, “Days”) ∧ swrlb:greaterThan(?h, 1) ∧ swrlb:lessThan(?h, 3) ∧ sqwrl:makeSet(?s, ?d) ∧ sqwrl:groupBy(?s, ?d) ∧ sqwrl:size(?no_of_days, ?s) ∧ swrlb:equal(?no_of_days, 7) -> sqwrl:select(?u, ?h, ?no_of_days)
5	Extremely Active	User(?u) ∧ isContextOf(?u, ?PAC-HLC) ∧ swrlb:equal(?PAC-HLC, “Exercising”) ∧ hasStartTime(?PAC-HLC, ?starttime) ∧ hasEndTime(?PAC-HLC, ?endtime) ∧ temporal:duration(?h, ?starttime, ?endtime, “Hours”) ∧ temporal:duration(?d, ?starttime, ?endtime, “Days”) ∧ swrlb:greaterThan(?h, 1) ∧ swrlb:lessThan(?h, 3) ∧ sqwrl:makeSet (?s, ?PA-HLC) ∧ sqwrl:groupBy(?s, ?PA-HLC) ∧ sqwrl:size(?Exercise_per_day, ?s) ∧ swrlb:equal(?Exercise_per_day, 2) -> sqwrl:select(?u, ?h, ?Exercise_per_day)
6	Meal Frequency	User(?u)∧ hasActivity(?u, ?Act) ∧ swrlb:equal(?Act, ”Eating”) ∧ hasStartTime(?Act, ?starttime) ∧ hasEndTime(?Act, ?endtime) ∧ temporal:duration(?d, ?starttime, ?endtime, “Days”) ∧ sqwrl:makeSet(?s, ?d) ∧ sqwrl:groupBy(?s, ?d) ∧ sqwrl:size(?no_of_days, ?s) ∧ swrlb:equal(?no_of_days, 1) ∧ sqwrl:makeSet(?Actset, ?Act) ∧ sqwrl:groupBy(?Actset, ?p) ∧ sqwrl:size(?freq, ?Actset) ∧ swrlb:greaterThan(?freq, 2) -> sqwrl:select(?u, ?freq,?no_of_days)

## Abbreviations

The following abbreviations are used in this manuscript:

MMCO	Mining Minds Context Ontology
LLC	Low-level Context
HLC	High-level Context
PA-HLC	Physical Activity High-level Context
N-HLC	Nutrition High-level Context
C-HLC	Clinical High-level Context
BP	Blood Pressure
BG	Blood Glucose
VF	Vertical Fusioning
HF	Horizontal Fusioning
